# Preventing Sepsis in Maintenance Dialysis: Is Adjudication of CRBSI Necessary?

**DOI:** 10.34067/KID.0000000000000407

**Published:** 2024-04-25

**Authors:** Afsheen Afzal, Jeffrey Silberzweig

**Affiliations:** 1 Division of Nephrology and Hypertension, Weill Cornell Medical College, New York, New York; 2 The Rogosin Institute, New York, New York

**Keywords:** clinical nephrology, dialysis access, ESKD, hemodialysis adequacy, vascular access

Of the 113,309 individuals who entered the United States Renal Data System as patients on incident in-center hemodialysis in 2021, 85% had central venous catheters (CVCs).^1^ Of the 462,539 patients receiving maintenance hemodialysis, 23% used a catheter for vascular access despite the accepted fact that CVCs are the least preferred vascular access. Arteriovenous fistulas (AVFs) have a longer functional life and lower risk of complications, including infections.^1–5^ Compared with patients with an AVF or arteriovenous graft, patients undergoing hemodialysis using CVCs have 2–3 times greater risk of blood stream infections (BSIs), hospitalizations, and mortality.^2,3^ The consequences of catheter-related BSIs (CRBSIs) extend beyond the immediate health effect because they are associated with longer hospital stays, increased health care costs, and a considerable burden on both the patients and the health care system.

Infections are the second leading cause of mortality among patients treated by maintenance hemodialysis with sepsis accounting for 6% of deaths in this population.^1^ Hemodialysis-CRBSIs represent a significant risk of sepsis, a dysregulated response to infection. Sepsis causes high rates of morbidity in addition to mortality, including complications, such as endocarditis and osteomyelitis. Prevention of sepsis is critical to reducing these complications. Prompt identification of infections is the critical first step allowing for timely intervention and reduced complications in this vulnerable population.

Another important tenet of the management of BSIs and of sepsis is source control: removing the source of the infection. Patients receiving maintenance dialysis must maintain vascular access sites often for many years, and repeated catheterization of central veins leads to stenosis and failure of distal access points. If a patient presents with BSI unrelated to a dialysis catheter, the catheter can be preserved, leading to continued use of a vascular access site. It is of paramount importance that all providers caring for a patient with BSI share an understanding of the source of that infection so that catheters can be removed when they are the source of infection but maintained when they are not.

Prevention of CRBSIs is an important quality initiative in all dialysis centers. Despite aggressive efforts to reduce the use of CVCs, they remain prevalent. Other infection prevention strategies include meticulous catheter care, proper hand hygiene, and use of sterile techniques during catheter insertion and manipulation. Regular monitoring and identification of CRBSIs are essential components of patient safety in hemodialysis units. Quality programs use patterns of CRBSI to determine when interventions, such as staff education, may be warranted.

To support clinicians in their efforts to improve the quality of care delivered to patients requiring maintenance dialysis, the Federal agencies tasked with overseeing their care have led the effort to decrease CVC utilization, enhance infection control measures in dialysis facilities, and decrease the incidence of CRBSI. The Fistula First initiative introduced in the early 2000s by the Centers for Medicare & Medicaid Services (CMS) led to a significant increase in AVFs.^4^ CMS has included measures related to AVF and CVC rates since the introduction of the ESRD Quality Incentive Program (QIP). Despite these measures, the percentage of patients initiated on hemodialysis using CVCs remains high.^1,4^ In response, CMS has removed the measure related to the proportion of fistulas in a dialysis facility from the QIP to enable patients to receive arteriovenous grafts when appropriate facilitating removal of CVCs. The Centers for Disease Control and Prevention conducts surveillance for BSIs among patients receiving hemodialysis through the National Healthcare Safety Network, which is also included in the ESRD QIP.^5^

In this issue of *Kidney360*, Catiwa *et al.*^6^ present an analysis of a clinical adjudication process on hemodialysis-CRBSI reporting on the basis of a *post hoc* analysis of the cluster randomized REDUcing the burden of Catheter ComplicatTION trial, involving 42 kidney centers in Australia and New Zealand. This very important work increases our understanding of CRBSIs by clarifying the relationship between the clinical definition of CRBSI and that of an outside adjudication panel. The study highlights the difficulty created by differing definitions for classifying BSIs as catheter related, which can result in misclassification of the outcomes because of the variability in interpretation of the results and observer bias. In this study, adjudication took place at multiple levels by independent nephrologists not affiliated with the REDUcing the burden of Catheter ComplicatTION clinical trial team. Inter-rater reliability was assessed using the Gwet agreement coefficient, which strengthens the study compared with the use of the more conventional kappa statistic. While adjudication can be used in clinical trials, its use outside of the research setting would be cost prohibitive and would likely take too long to have clinical utility.

The study makes important progress toward a gold standard definition of CRBSIs, which is needed for accurate surveillance and reporting of CRBSIs. Despite efforts to achieve consensus by the Centers for Disease Control and Prevention, the Infectious Diseases Society of America (IDSA), and the Kidney Disease Outcomes Quality Initiative, criteria are not widely accepted. Universal criteria are essential for dialysis facilities to evaluate their own infection rates and outcomes and to compare their performances with one another and against established standards, thereby facilitating quality improvement initiatives. For dialysis facility quality improvement programs to succeed, they need accepted goals for achievement. Equally importantly, patients need access to clear information so that they can compare facilities and be in the best position to choose their providers.

This study suggests that a modification of the IDSA definition of hemodialysis-CRBSI with demonstrated concordance between reported and adjudicated hemodialysis-CRBSI events could be the answer to this important problem because the validity of benchmarking without the need for time-consuming independent clinical adjudication facilitates both patient care and quality measurement. To verify the validity of benchmarking without adjudication, further studies should be conducted on a larger population of patients with hemodialysis catheters to ensure the validity of the modified IDSA definition of hemodialysis-CRBSI is retained.
